# Identification, Classification, and Growth of Moa Chicks (Aves: Dinornithiformes) from the Genus *Euryapteryx*

**DOI:** 10.1371/journal.pone.0099929

**Published:** 2014-06-12

**Authors:** Leon Huynen, Brian J. Gill, Anthony Doyle, Craig D. Millar, David M. Lambert

**Affiliations:** 1 Environmental Futures Centre, Griffith University, Nathan, Qld, Australia; 2 Auckland War Memorial Museum, Auckland, New Zealand; 3 Radiology with Anatomy, Faculty of Health and Medical Sciences, University of Auckland, Auckland, New Zealand; 4 Allan Wilson Centre for Molecular Ecology and Evolution, School of Biological Sciences, University of Auckland, Auckland, New Zealand; Natural History Museum of Denmark, Denmark

## Abstract

**Background:**

The analysis of growth in extinct organisms is difficult. The general lack of skeletal material from a range of developmental states precludes determination of growth characteristics. For New Zealand's extinct moa we have available to us a selection of rare femora at different developmental stages that have allowed a preliminary determination of the early growth of this giant flightless bird. We use a combination of femora morphometrics, ancient DNA, and isotope analysis to provide information on the identification, classification, and growth of extinct moa from the genus *Euryapteryx*.

**Results:**

Using ancient DNA, we identify a number of moa chick bones for the species *Euryapteryx curtus*, *Dinornis novaezealandiae*, and *Anomalopteryx didiformis*, and the first chick bone for *Pachyornis geranoides*. Isotope analysis shows that ∂^15^N levels vary between the two known size classes of *Euryapteryx*, with the larger size class having reduced levels of ∂^15^N. A growth series for femora of the two size classes of *Euryapteryx* shows that early femora growth characteristics for both classes are almost identical. Morphometric, isotopic, and radiographic analysis of the smallest *Euryapteryx* bones suggests that one of these femora is from a freshly hatched moa at a very early stage of development.

**Conclusion:**

Using morphometric, isotopic, and ancient DNA analyses have allowed the determination of a number of characteristics of rare moa chick femora. For *Euryapteryx* the analyses suggest that the smaller sized class II *Euryapteryx* is identical in size and growth to the extant Darwin's rhea.

## Background

In depth analysis of growth in ancient animals is often limited due to the scarcity and degraded nature of skeletal material or tissues of different ages. Similarly, the rare occurrence of different aged bones for New Zealand's extinct ratite moa (Aves: Dinornithiformes) has made any analysis of moa growth difficult [1], [2], [3].

Adult moa ranged in size from less than 20 kg for the small coastal moa *Euryapteryx curtus curtus* to over 200 kg for the South Island giant moa *Dinornis robustus* [2]. The identification of species within the *Euryapteryx* genus has been particularly difficult. Latest data suggest the existence of a small subspecies (*E. curtus curtus*) limited to New Zealand's North Island, and a larger subspecies (*E. curtus gravis*) found only in New Zealand's South Island [4].

How moa grew is largely unknown with most published work comparing moa to the growth characteristics of their extant relatives [2], [3]. Relatively recent work analysing cortical growth marks in moa limb bones suggest that, unlike their modern relatives, moa had a particularly long pre-adult growth period [5].

We analyse moa growth using a number of rare moa chick femora, currently housed at New Zealand's Auckland Museum and kindly made available to us. The museum houses a significant number of samples of moa chick bones from sand-dune sites in New Zealand's upper North Island, especially from the Karikari Peninsula/Doubtless Bay area, including Tokerau Beach. Adult bones from these sites have been attributed to three moa species with most being derived from *E. curtus curtus* [2], [6], [7].

To date, only one embryonic moa has been identified to species, where bones associated with an egg were shown to belong to the heavy-footed moa *Pachyornis elephantopus* [2], [8]. As bones of developing chicks, which often lack identifying characters, are particularly difficult to identify [1], [2] we use a minimally destructive technique to genetically assign differently sized immature moa femora to the species level. We then use bone morphometrics to present a growth series of chick femora for *Euryapteryx*. In addition, we present isotope and radiographic data for the smallest moa femora to determine whether these may have derived from unhatched eggs. The isotope data has also allowed us to further explore the status of two subspecies proposed for moa from the genus *Euryapteryx* [9].

## Results

**Figure 1:**
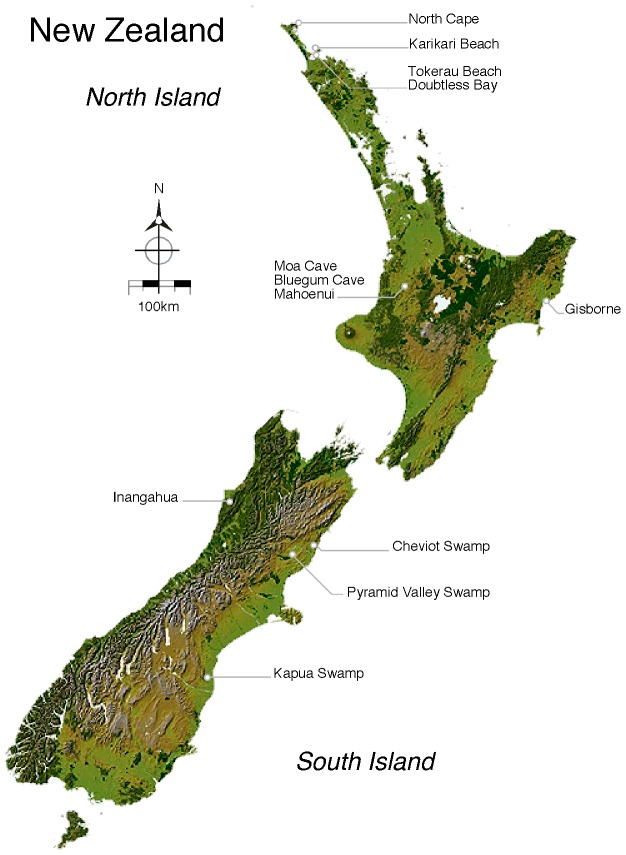
Moa sample locations. The locations of moa samples used in this work are shown.

**Figure 2:**
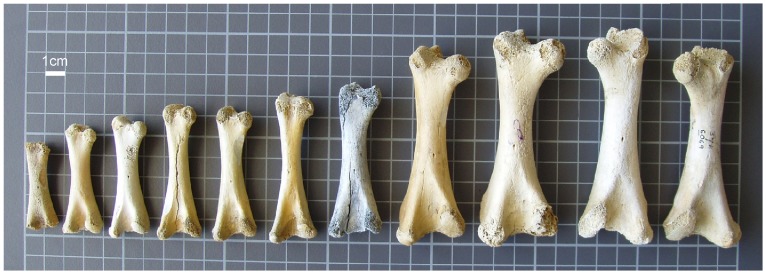
Examples of moa chick femora. From left to right: LB5990, LB8295, LB6070, LB6261d, LB6284, LB6261c, LB12961, LB6285b, LB6657, LB6071, LB6069. All are left femora except the two at far right. DNA analysis suggests all are from Euryapteryx except for LB6261c which was identified as Dinornis. Gridlines are at 10 mm intervals.

We successfully amplified a relatively short (∼70 bp) hypervariable mitochondrial DNA fragment from 29 of 32 immature bones sampled from various locations in New Zealand (Tables 1,2; Figures 1,2). Femur LB6261c was identified as belonging to *Dinornis novaezealandiae* and is 72 mm long (Table 2, Figure 2). Bones of a late-term embryonic moa (identified as *Pachyornis elephantopus*) were recovered from inside an egg in 1866 [2], [8]. The egg was 226 mm long and 155 mm wide with the embryonic femur being approximately 48 mm long (with ends restored). An egg found at Kaikoura and attributed to *Dinornis* was shown to be 240 mm long, and by proportion, its embryo (if at the same stage as the *Pachyornis* egg) would have had a femur approximately 51 mm long. Therefore the size of LB6261c suggests it was from a recent hatchling. Further evidence for the extreme immaturity of this femur is the lack of caudal tuberosities on the femur shaft, a feature of the femur that separates *Dinornis* from the emeid moas [1], [2]. Turvey and Holdaway (2005) [3] described ‘postnatal’ bones of *Dinornis* and showed that femora at growth-stage 1 began at 156 mm in length with their youngest stage 1 individual having an estimated weight of 15.8 kg. Thus their sample included only well-grown chicks and did not include hatchlings. The single *Pachyornis geranoides* femur: At 82 mm long, LB7976 (Table 2) is likely to be from a well-developed chick, since this moa species is relatively small. Although difficult to determine due to erosion, an excavation at the proximal end of the bone may be the pneumatic fossa that characterises this species [1], [2]. LB7976 is the only known chick bone of this species. Four *Anomalopteryx didiformis* femora were identified; AIM LB6666c, AIM LB6261a-b, and AIM LB6285a (Table 2), all derive from Doubtless Bay, and are the first chick bones to be identified (by DNA) for this species, as well as being the first record of this species from Doubtless Bay. For *Euryapteryx curtus* 22 femora specimens were identified by DNA, and include the 11 smallest femora (see Figure 2). These form the first large sample of chick bones attributable with certainty to this species, and were used for detailed morphological, radiographic, and isotopic analyses. The sequence targeted allows discrimination of all moa species and identifies two distinct genetic haplotypes for *Euryapteryx*. The haplotypes can be separated by a single SNP that associates class I *Euryapteryx* with thick eggshells (0.98 mm - 1.60 mm) and class II *Euryapteryx* with thin eggshells (0.74 mm–0.98 mm) [6]. The distribution of the two *Euryapteryx* classes (I and II) closely mimics the distribution proposed for subspecies *E. curtus gravis* and *E. curtus curtus* respectively [4].

**Table 1:** Moa sequences.

Mt hpt.	Sequence
E1	-----G--------T-A--------------
E5	-----G--------T-A---C---A------
E14	---T-G--------T-A---C--------C-
D15	—T—R-C-CC:---C:---C—T—CTC—
P1	-C-------C-A-----AC----T-------
A1	-C-TC—G---:-----AT------------
Cons	CTCCTAAACTACCCCTT::TTCACGCTCTTC

Mitochondrial haplotypes (*Mt hpt*.) found and their sequences are shown. - identical base to consensus (*Cons*) sequence. : - gap. Haplotypes are numbered as reported in [7]. *E - Euryapteryx curtus, D - Dinornis novaezealandiae, P - Pachyornis geranoides, A - Anomalopteryx didiformis.*

**Table 2:** Identification and morphometrics of moa chick femora from Auckland Museum.

Museum ID	Side	L	W	Weight (kg)	*Species* (class)	*Mt hpt*
LB12960	R	c.47.0	8.5	0.470	*Eu* (II)	*E14*
LB5990	L	c.48.0	8.9	0.498	*Eu* (II)	*E14*
LB8295	L	53.5	9.7	0.674	*Eu* (I)	*E1*
LB6261e	L	58.6	8.8	0.869	*Eu* (I)	*E1*
LB6070	L	59.0	9.9	0.885	*Eu* (II)	*E14*
LB13978	L	60.5	10.2	0.949	*Eu* (I)	*E1*
LB6259	R	60.7	10.0	0.958	*Eu* (II)	*E14*
LB6284	L	64.5	10.5	1.135	*Eu* (II)	*E14*
LB6261d	L	65.1	9.5	1.164	*Eu* (I)	*E1*
LB6285d	L	65.6	9.7	1.189	*Eu* (II?)	*E14?*
LB6285c	L	68.5	11.2	1.342	*Eu* (II)	*E14*
LB6666c	R	70.2	12.1	1.437	*Ad*	*A1*
LB6261c	L	72.0	8.7	1.541	*Dn*	*D15*
LB12961	L	73.1	12.2	1.608	*Eu* (I)	*E1*
LB6666b	L	77.9	13.2	1.920	*Eu* (I)	*E1*
LB7976	R	81.7	12.7	2.192	*Pg*	*P1*
LB6261b	L	83.7	12.8	2.345	*Ad*	*A1*
LB6260	L	86.0	13.5	2.529	?	*?*
LB6261a	L	86.5	12.4	2.570	*Ad*	*A1*
LB6072	L	89.7	14.0	2.843	*Eu* (II)	*E14*
LB6285b	L	94.1	14.8	3.249	*Eu* (II)	*E14*
LB6601	L	94.3	13.4	3.268	*Eu* (I)	*E5*
LB6069	R	96.9	15.2	3.526	*Eu* (II)	*E14*
LB6285a	L	100.9	16.3	3.946	*Ad*	*A1*
LB6657	L	101.4	15.6	4.001	*Eu* (I)	*E1*
LB6071	R	103.4	16.4	4.225	*Eu* (II)	*E14*
LB6832	L	114.3	17.0	5.585	*Eu* (II)	*E14*
LB6680	L	120.1	18.0	6.411	*Eu* (II)	*E14*
LB6711	L	127.6	19.4	7.589	?	*?*
LB6266	L	134.0	21.4	8.697	?	*?*
LB6666a	R	134.0	20.7	8.697	*Eu* (II)	*E14*
LB6257	R	142.1	21.7	10.24	*Eu* (I)	*E5*

All bones are from Karikari Peninsula and Doubtless Bay, Northland, except LB7976 (North Cape) and LB6601 (Gisborne). Samples were identified by amplification and sequencing of a ∼30 bp fragment of mitochondrial hypervariable region I (HVRI). *Euryapteryx* class I and class II correspond to *Euryapteryx* sequences shown previously to be associated with thick and thin shells respectively [7]. *Eu – Euryapteryx curtus , Ad - Anomalopteryx didiformis, Pg - Pachyornis geranoides, Dn - Dinornis novaezealandiae. Mt hpt. -* Mitochondrial haplotype sequences are shown in Table 1.

L =  total length, W =  mid shaft width. Weight was calculated using femur length according to the equation: Mass (kg)  =  (L/61.64)^2.7855^ [11].

**Figure 3:**
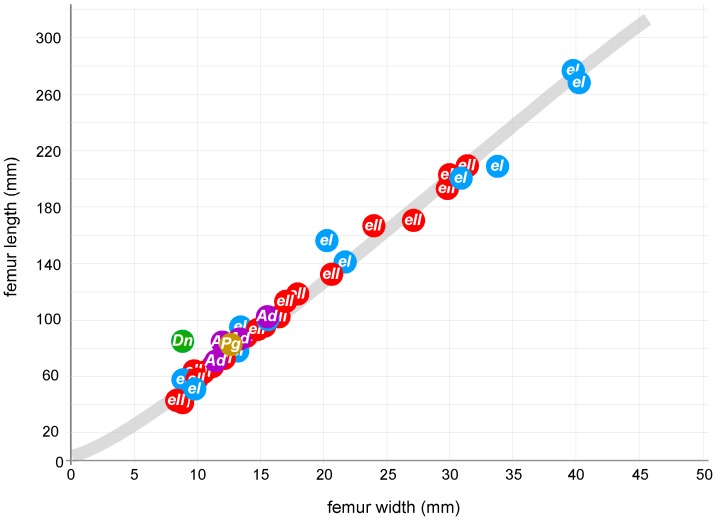
Femoral growth series for chick bones from *Euryapteryx curtus*. Graph showing femur length vs width (mm). Blue - *Euryapteryx* class I, *eI*; Red - *Euryapteryx* class II, *eII*; For comparison, chick bones from other moa species identified are also shown. Green - *D. novaezealandiae*, *Dn*; Purple - *A. didiformis*, *Ad*; Brown - *P. geranoides*, *Pg*. A line of best fit is shown in grey.

The length and width of each femur was recorded for both *Euryapteryx* classes and compared to obtain a growth series (Figure 3). No difference could be found in early femora growth characteristics between the two *Euryapteryx* classes. Morphometric and mass calculations were carried out on the smallest *Euryapteryx* femur (LB12960; belonging to class II *Euryapteryx*) to determine whether this femur may have been from an unhatched egg. Two femur-based equations exist that are commonly used to determine avian mass; one using least shaft circumference provided by [15] and one using total femur length [11] (see methods). Although both methods are relatively accurate for the determination of mass of an avian adult, mass calculation using femur length has been shown to be more accurate for developing birds [16]. Using femur length, estimated at c. 47 mm (measured at 44 mm but adding 3 mm to allow for missing ends) the mass of the LB12960 individual was calculated to be only 470 g. For Darwin's rhea, very similar to class II *Euryapteryx* in adult size (15–28.6 kg) and eggshell thickness (0.73–1.1 mm) [17] newly hatched individuals range in weight from 0.327–0.491 kg with an average weight of 0.426 kg [18]. This suggests that femur LB12960 may have derived from a moa embryo, but is likely to be from a very young newly hatched chick.

**Figure 4:**
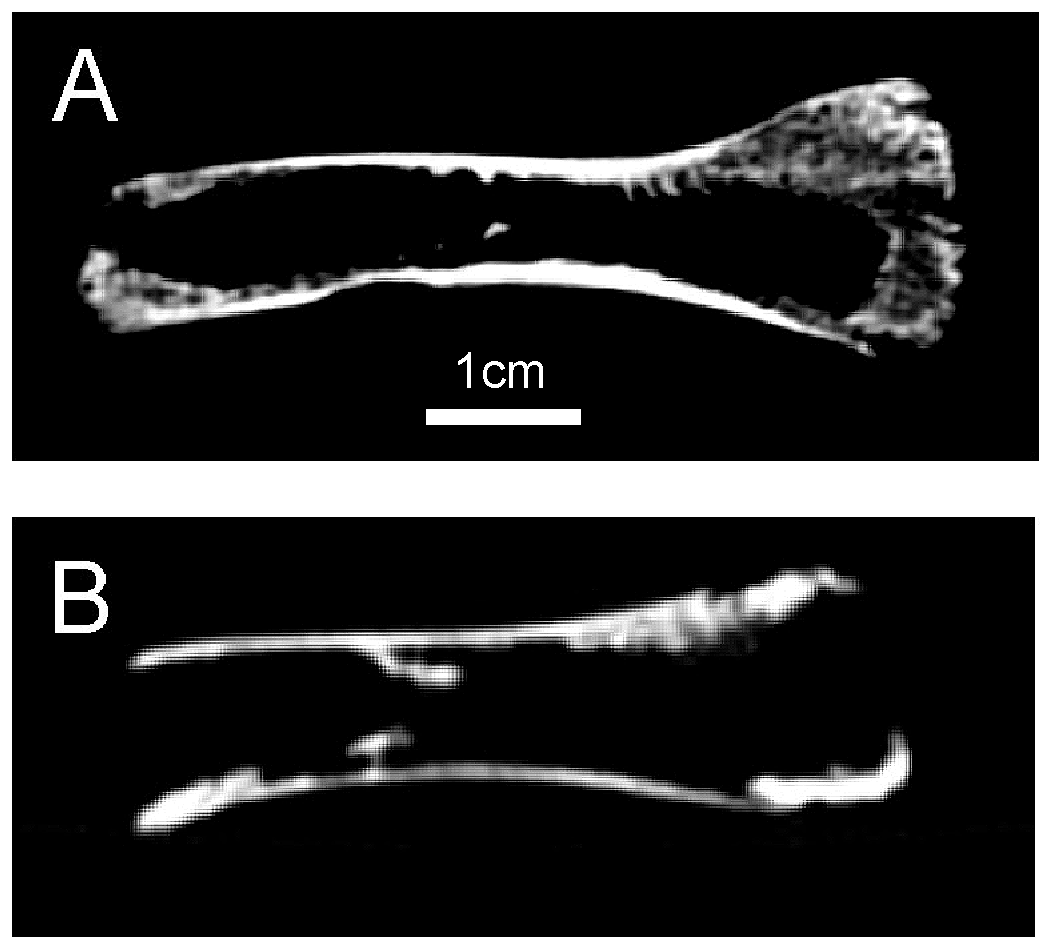
Radiographic analysis of *Euryapteryx* class II femora. **A**. Chick LB13978: more mature form with intact bone end on right, consistent with chick of a few weeks age where cartilage cone has migrated down shaft. **B**. Chick LB12960: immature bone with open end on right, consistent with cartilage cone present up to growth plate as occurs with embryo and neonate.

Further analysis of the developmental stage of the smallest *Eurypateryx* femora was carried out by radiography. Six bones were analysed, of which four had essentially intact bone of variable thickness at the ends (Table 3). These ends correspond to the metaphyses of the femora adjacent to the growth plates, the epiphyses having been lost or separated. The ends of intact bones suggest that the cartilage ‘cones’ described by [19] had already migrated away from the bone ends and growth plates. This is a feature found in their study of rheas only after the birds were of 3 weeks maturity or more. It is therefore reasonable to assume that these four chicks were a few weeks old at least. Only two of the moa chick bones had complete defects in the metaphyses, identifying them as neonatal or embryonic (Figure 4). One of these (LB12960) has a slightly larger defect than the other and subjectively has less trabecular bone overall than any of the others. These data further suggest that this bone may have come from a late stage embryo or newly hatched chick, in concordance with the nitrogen isotope (below) and morphometric findings (Table 2).

**Table 3:** Radiographic analysis of moa chick femora.

Museum ID	Cross section shape	Presumed site	Intact cortex	Trabecular bone (minimum mm)	Signs of cone to growth plate	Comment
LB6261e	flat	proximal	Y	3	N	Thicker trabecular bone next to “head”.
LB12960	flat	proximal	N	0	Y	Complete defect proximal bone end, possibly embryonic
LB8295	flat	proximal	Y	3	N	Tiny eccentric defect
LB13978	flat	proximal	Y	6	N	No defect
LB5990	flat	proximal	N	0	Y	Central defect
LB6070	flat	proximal	Y	3	N	No defect

Analyses were carried out mainly on the proximal femur end, although the distal ends are similar [19].

**Figure 5:**
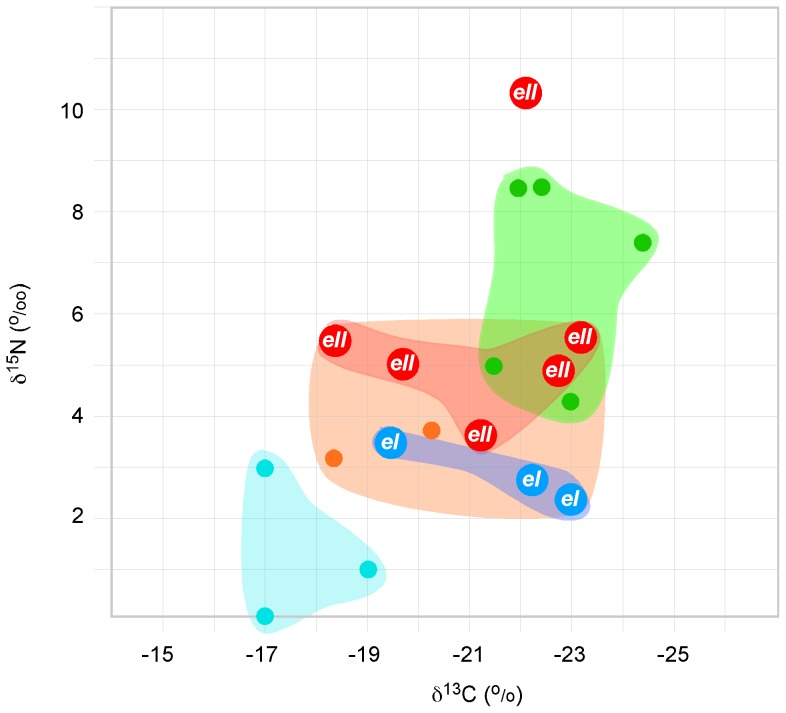
Isotope analysis of selected moa bones. Moa bone samples from different species (Table 3) and environments were subjected to isotopic analyses. Light blue - cave samples; Orange - Dune samples; Green - Swamp samples; Blue - *Euryapteryx* class I, *eI*; Red - Euryapteryx class II, *eII*; The isolated *eII* sample is the possible newly hatched *Euryapteryx* class II individual.

Additional analyses were carried out on the smallest femora using isotope counts in the hope that femora from unhatched individuals, by feeding on yolk, have different ∂^15^N and/or ∂^13^C levels to femora from hatched chicks feeding on insects. To account for habitat or species-specific biases, isotope levels were determined for several moa species from different environments including sand-dune, cave, and swamp (Table 4, Figure 5). No species-specific differences in isotope levels were found among the few samples that were measured. However, moa bone samples from caves had very low ∂^15^N values (−0.1–2.90 parts per million (^o^/oo)), while those from swamps had ∂^15^N levels of 4.2–8.6^ o^/oo and sand-dune samples ranged in ∂^15^N from 3.1–5.5 ^o^/oo, values that are likely to be indicative of the specific environment (Table 4, Figure 5). Isotopic analysis showed relatively low values of ∂^13^C (18.3–23.1 ^o^/o) in all *Euryapteryx* bones suggesting that individuals of this moa species preferred more open habitats and, as expected for New Zealand, a C3-based diet [20].

**Table 4:** Isotope analysis of moa bones from different environments.

Sample ID	Elemental % Comp		Isotopic Delta		*Species* (class)	Site	Location
	(C)	(N)	(C13)	(N15)			
AIM LB12960	17.0	3.2	−22.2	10.3	*Eu* (II)	sand-dune	Karikari Beach
AIM LB5990	11.4	2.8	−22.8	4.9	*Eu* (II)	sand-dune	Tokerau Beach
AIM LB8295	12.6	2.9	−22.3	2.7	*Eu* (I)	sand-dune	Tokerau Beach
AIM LB6261e	13.4	3.1	−23.0	2.2	*Eu* (I)	sand-dune	Tokerau Beach
AIM LB6070	5.1	0.5	−23.1	5.4	*Eu* (II)	sand-dune	Tokerau Beach
AIM LB13978	7.8	1.6	−19.5	3.6	*Eu* (I)	sand-dune	Tokerau Beach
CM Av21547	4.4	0.9	−19.1	1.0	*Ad*	cave	Bluegum Cave
CM Av34550	5.4	0.9	−17.1	2.9	*Ad*	cave?	Inangahua
CM Av22420	5.0	0.9	−17.1	−0.1	*Dn*	cave	Moa Cave
AIM LB6292	9.0	2.4	−20.3	3.8	*Dn*	sand-dune	Tokerau Beach
AIM LB7070	4.1	0.6	−18.3	3.1	*Dn*	sand-dune	Doubtless Bay
AIM LB6425	7.4	1.9	−18.3	5.5	*Eu* (II)	sand-dune	Tokerau Beach
AIM LB6422	8.1	2.1	−19.8	5.0	*Eu* (II)	sand-dune	Tokerau Beach
AIM LB6423	9.7	2.8	−21.2	3.5	*Eu* (II)	sand-dune	Tokerau Beach
CM SB301	11.2	3.5	−21.6	5.1	*Pel*	swamp	Cheviot Swamp
CM Av15036	12.0	3.5	−22.6	8.6	*Pel*	swamp	Pyramid Valley Swamp
CM Av8317	11.1	3.3	−22.0	8.6	*Ecr*	swamp	Pyramid Valley Swamp
CM Av9130	11.7	3.5	−23.1	4.2	*Ecr*	swamp	Kapua Swamp
CM Av8312	12.4	3.8	−24.3	7.4	*Ecr*	swamp	Pyramid Valley Swamp

% composition for carbon (C), nitrogen (N) and isotopic delta for ^13^C (C13) and ^15^N (N15) are shown. *Eu – Euryapteryx curtus* (class I or II), *Ad - A. didiformis*, *Dn - D. novaezealandiae*, *Pel - P. elephantopus*, *Ecr - Emeus crassus*.

As almost all *Euryapteryx* chick bones in this study were obtained from the same area, climate and habitat differences are unlikely to be a source of isotope variation. Interestingly, ∂^15^N levels between the two *Euryapteryx* classes showed class I ∂^15^N values were generally low and ranged from 2.2–3.6^ o^/oo (n = 3, mean  = 2.83, SD = 0.58) while class II ∂^15^N values ranged from 3.5–5.5^ o^/oo (n = 6, mean  = 4.86, SD = 0.71). Although the sample numbers are low, the class I and class II∂^15^N values proved to significantly different; *p* = 0.0115, Table 4, Figure 5). An unusually high ∂^15^N level (10.3 ^o^/oo) was found for the smallest *Euryapteryx* bone, AIM LB12960 (Table 4, Figure 5).

## Discussion

Morphometrics of femora from each variant showed very similar growth curves at the early stages of development. For the closely related emu and ostrich it has been shown that cortical bone thickness remains constant for the first two months after hatching, with the highest radial growth rate occurring 7–14 days posthatch [21]. Similarly for *Euryapteryx*, extrapolation of the growth curve to 0 suggests that at the very early stages of development, femur width increased proportionately more than femur length (Figure 3). For class II *Euryapteryx*, femur growth is linear until femur length reaches approximately 150 mm.

Isotope analysis gives an indication of diet, climate, and habitat. Carbon 13 (^13^C) levels in bone give an indication of whether the plants ingested utilized a C3 or C4 photosynthetic pathway, while Nitrogen 15 (^15^N) levels provide information on the organisms trophic level [22]. In general, bone ∂^13^C values become more negative when animals feed in open shrubland, as opposed to vegetation from closed canopy or forested areas [2], [20], [23]. ∂^15^N levels are an indication of not only trophic level but can also be indicative of habitat, climate, and salinity, with high ∂^15^N values being more characteristic of individuals found in more saline, hot, dry environments [24]. The Isotope data we obtained provides further evidence that the two genetic variants of *Euryapteryx curtus* are likely to represent subspecies. One of these was likely to be a small sized moa that was associated with thin eggshells. The other was probably a larger sized moa associated with thick eggshells [8]. The smallest *Euryapteryx* class II bone sample (LB12960) returned a very high ∂^15^N value. High ∂^15^N values are generally associated with high trophic levels. It has been found, for sharks at least, that embryos have elevated ∂^15^N levels compared to their mothers [25]. Furthermore ∂^15^N values similar to those found in sample LB12960 have been obtained for egg yolk from insect-fed (high protein) chickens [26] suggesting, in accordance with the morphometric and radiographic data, that this femur may have come from inside an egg or from a newly hatched individual.

Newly hatched ostrich and emu chicks increase in body mass by at least 50% per month, and reach adult size within one year [21]. For *Euryapteryx*, this process is likely to be much slower with the smaller *Euryapteryx curtus curtus* (North Island subspecies; adult weight ∼20 kg; [2] reaching maturity after 4 years and the larger *Euryapteryx curtus gravis* (mainly South Island subspecies; adult weight ∼80 kg; [2]) not reaching adulthood for over 9 years [4]. Assuming *E. c. curtus* and *E. c. gravis* neonates average 0.43 kg [18] and 0.95 kg respectively (latter weight based on average ostrich hatch weight; [27]), this would suggest a growth rate for *Eu. curtus curtus* of ∼10% per month and *Eu. curtus gravis* a very slow ∼5% per month. For this reason, moa chicks were likely to be highly dependent on adult(s) support for a significant period of time. This is somewhat unusual for ratites where extant ratites such as the kiwi, emu, ostrich, and rhea are considered precocious and require little adult support beyond the initial growth stages [17]. In direct contrast to emu, ostrich, and rhea which are subject to predation by goannas, hyenas, and large cats respectively [28] it is perhaps fortunate that prior to the arrival of humans, moa had very few natural predators [2]. This scarcity of natural predators may have allowed moa to survive its protracted early stages of development.

We use a variety of analytical methods to gain information on rare chick bones from New Zealand's moa. The analyses have allowed the identification of the first chick bone for *P. geranoides*, the determination of early growth for moa from the genus *Euryapteryx*, the characterization of a very rare newly hatched *Euryapteryx* individual, and have also provided futher evidence for the existence of possible subspecies within this genus.

## Materials and Methods

### Moa bone samples

All moa bone samples were kindly loaned by the Auckland War Memorial Museum (AIM) and Canterbury Museum (CM). Chick bones were identified from the Auckland War Memorial Museum (AIM) moa bone collection by their reduced size in relation to adult bones. Well preserved specimens with known collection locality were selected for sampling. Where possible the left femur was chosen. Permission to sample moa specimens was obtained from the respective museum curators. No permits were required for the described study, which complied with all relevant regulations.

### Morphometrics

Maximum femur length was determined by measuring parallel to the long axis of shaft, as shown in Figure 4Al of [9]. Femur width was measured at mid-point along the shaft, at right angles to the anterior-posterior plane with the femur head to one side as shown in Figure 5.III.D of [10]. Immature femora were measured irrespective of incompleteness due to a lack of fused epiphyses. Measurements were made to the nearest 0.1 mm using Vernier calipers. Moa weight was determined using total femur length where body mass (kg)  =  (femur length (mm)/61.64)^2.7855^ according to [11].

### Radiography

The smallest moa bones were subjected to computed tomography at high resolution using a multi-detector scanner (Siemens Emotion 16, Siemens Medical, Erlangen, Germany). Helical thin slices (0.6 mm, 90 mm field of view, 130 kilovolt peak, 60 milliampere seconds, 512×512 matrix, bone algorithm) were generated and reconstructed into 0.6 mm contiguous slices along and at right angles to the bone. These were viewed and measured on a PC using Siemens software (Syngo fastView, Siemens Berlin, 2009).

### Isotope analysis

Isotope analysis was carried out on selected bones at the Australian Rivers Institute, Griffith University, Australia using the following standards: Primary; N - Ambient air IAEA-305a, C - ANU sucrose, Elemental; Acetanilide Working; ‘Prawn’. Mass Spectrophotometry was carried out on a GV Isoprime spectrophotometer (Manchester UK) using a Eurovector EA 3000 inlet. Samples were taken from the outer (radial) layer where possible [12].

### DNA extraction and amplification

DNA was extracted, amplified, and sequenced using the mitochondrial primers mcrshFF and mcrshRR as outlined in [13]. DNA was extracted from approximately 50 mg of bone shavings by incubation overnight at 56°C with proteinase K, and then purified by phenol:chloroform extraction and silica bed binding using a Qiagen DNeasy Blood & Tissue Kit. DNA was eluted from the silica column and subjected to PCR using the mitochondrial control region primers mcrshFF and mcrshRR as outlined in [13] and visualised by agarose gel electrophoresis and ethidium bromide staining. Positive amplifications were sequenced using primer F6t as described in [13]. DNA results were obtained blind, where bone identity remained unknown until sequences were obtained.

### Ancient DNA procedures

DNA was extracted and amplified according to the criteria proposed by [14]. Ancient DNA was extracted in a dedicated ancient DNA laboratory at Griffith University, Australia, and amplified at a separate isolated modern lab facility. For sequence verification, several samples were replicated independently at the Massey University Ancient DNA facility, Albany, Auckland, New Zealand.
