# Correction: Identification, Classification, and Growth of Moa Chicks (Aves: Dinornithiformes) from the Genus *Euryapteryx*

**DOI:** 10.1371/journal.pone.0108995

**Published:** 2014-09-22

**Authors:** The PLOS ONE Staff

Table 1 is aligned incorrectly due to errors that occurred during the typesetting process. The correct version of Table 1 can be viewed below.

**Table 1:** Moa sequences. Mitochondrial haplotypes (*Mt hpt*.) found and their sequences are shown. - identical base to consensus (*Cons*) sequence.: - gap. Haplotypes are numbered as reported in [7]. *E - Euryapteryx curtus, D - Dinornis novaezealandiae, P - Pachyornis geranoides, A - Anomalopteryx didiformis*.

*Mt hpt.*	Sequence
*E1*	-----G--------T-A--------------
*E5*	-----G--------T-A---C---A------
*E14*	---T-G--------T-A---C--------C-
*D15*	--T--R-C-CC:---C:---C--T--CTC--
*P1*	-C-------C-A-----AC----T-------
*A1*	-C-TC--G---:-----AT------------
*Cons*	CTCCTAAACTACCCCTT::TTCACGCTCTTC
